# Supplementary material for: Epigenetic control of metabolic identity across cell types
Source: bioRxiv. 2024 Jul 24:2024.07.24.604914. Preprint. [Version 1] doi: 10.1101/2024.07.24.604914 (PMC11291179; doi:10.1101/2024.07.24.604914)
Supplement: Supplementary file 1 [file NIHPP2024.07.24.604914v1-supplement-1.pdf]

bioRxiv preprint doi: https://doi.org/10.1101/2024.07.24.604914; this version posted July 24, 2024. The copyright holder for this preprint (which was not certified by peer review) is the author/funder, who has granted bioRxiv a license to display the preprint in perpetuity. It is made available under aCC-BY 4.0 International license.

Epigenetic control of metabolic identity across cell types. Supplementary Data

Supplementary Table S1: The ChromGene state integrates active and repressive marks to obtain a gene-specific state instead of a locus state. 1-6 are active states, 7 corresponds to poised and 8-12 are repressive states.

Nb	*Name*	*Description*
1	strong trans enh	Strongly transcribed genes with enhancer marks H3K4me1 and H3K27ac, highest expressed
2	trans	Transcribed genes, highly expressed. Lower levels of H3K36me3 and H3K4me1 than strong trans enh
3	trans k36me3	Transcribed genes, moderately expressed, marked with H3K36me3. Relatively low levels of enhancer marks
4	trans enh	Transcribed genes, moderately expressed, marked with enhancer marks H3K4me1 and to a lesser extent H3K27ac. Relatively low levels of H3K36me3.
5	trans wk	Transcribed genes, lowly expressed, marked with enhancer mark H3K27ac and to a lower extent, H3K4me3. Relatively low levels of H3K36me3
6	ZNF	Zinc Finger-related genes, with a state marked by H3K36me3 and H3K9me3
7	poised	Lowly-expressed genes with high levels of H3K4me1, but low levels of H3K36me3.
8	PC repr wk	Lowly expressed genes with high levels of H3K4me1, weakly marked with polycomb repressive mark H3K27me3.
9	bivalent	Lowly expressed genes, marked with high level of repressive mark H3K27me3, and moderate levels of H3K4me1 and H3K4me3
10	quiescent	Lowly expressed genes, lack of significant histone marks
11	het	Generally unexpressed genes associated with heterochromatin.
12	PC repr	Generally, unexpressed genes, marked with polycomb repressive mark H3K27me3.

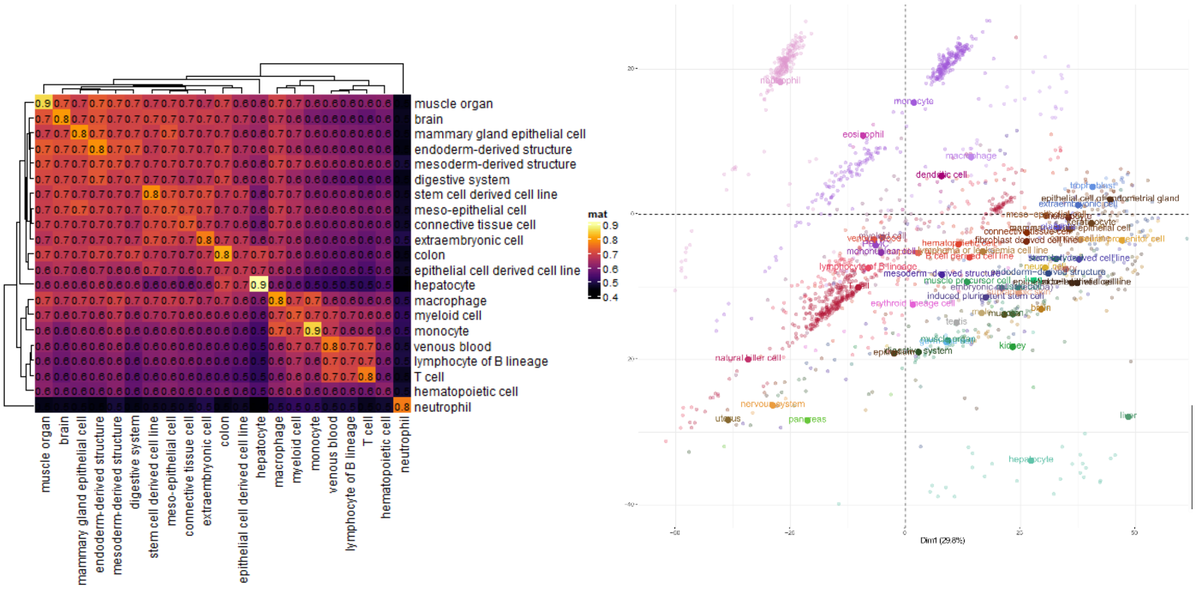

Supplementary Figure S1: Metabolic models capture the metabolic identity of tissues and cell types.

A) Hierarchical clustering of metabolic models by median Jaccard Similarity Indexes. A Jaccard Similarity Index (JSI) was computed for each pair of models. Then a median JSI was computed for each combination of cell types and tissues. The similarity within cell types and tissue tended to be larger

bioRxiv preprint doi: https://doi.org/10.1101/2024.07.24.604914; this version posted July 24, 2024. The copyright holder for this preprint (which was not certified by peer review) is the author/funder, who has granted bioRxiv a license to display the preprint in perpetuity. It is made available under aCC-BY 4.0 International license.

than between different cell types and tissues. B) Principal component analysis of the metabolic gene expression data. Neutrophils are separated from the other blood cell samples. Non-blood cells are separated from the blood cells along PC1. The hepatocyte and liver samples like neutrophils also form a distinct cluster but at the other extremity of PC1 and PC2.

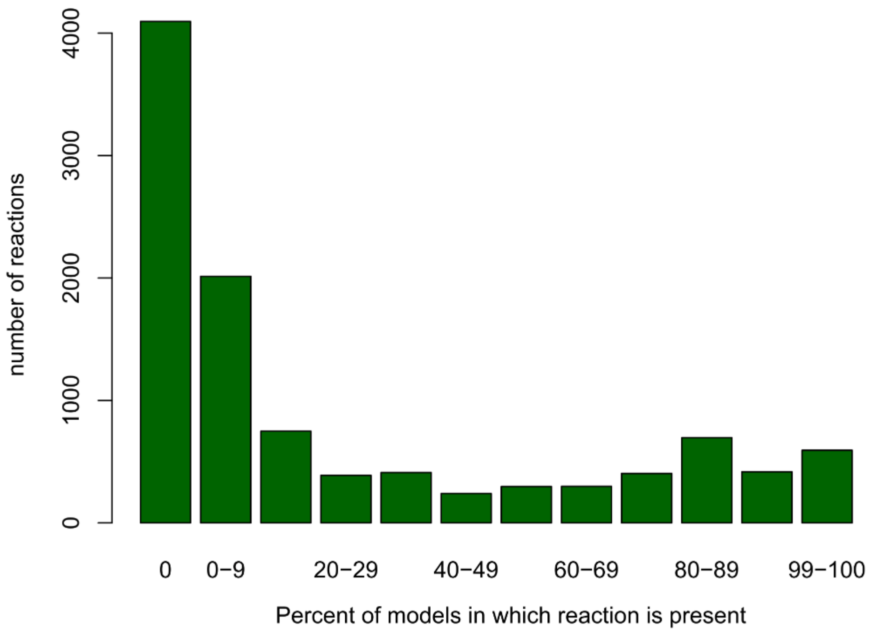

Supplementary Figure S2: Distribution of the number of models in which each reaction is present across the 1,555 metabolic models.

Supplementary Table S2: Number of reactions per pathway that were not included in any model.

Pathways	***Number***	***Pathways***	***Number***	***Pathways***	***Number***
Exchange/demand reaction	1231	*Dietary fiber binding*	12	*Glycolysis/gluconeogenesis*	4
Transport, extracellular	1022	*Methionine and cysteine metabolism*	12	*N-glycan synthesis*	4
Drug metabolism	442	*N-glycan degradation*	12	*Urea cycle*	4
Peptide metabolism	241	*Folate metabolism*	11	*Cholesterol metabolism*	3
Fatty acid oxidation	129	*Nucleotide interconversion*	11	*Glycosphingolipid metabolism*	3
Transport, mitochondrial	74	*O-glycan metabolism*	11	*Limonene and pinene degradation*	3
Tyrosine metabolism	66	*Transport, nuclear*	11	*Oxidative phosphorylation*	3

bioRxiv preprint doi: https://doi.org/10.1101/2024.07.24.604914; this version posted July 24, 2024. The copyright holder for this preprint (which was not certified by peer review) is the author/funder, who has granted bioRxiv a license to display the preprint in perpetuity. It is made available under aCC-BY 4.0 International license.

Miscellaneous	57	*Glycine, serine, alanine, and threonine metabolism*	10	*Pyruvate metabolism*	3
Keratan sulfate degradation	55	*Pentose phosphate pathway*	9	*Vitamin E metabolism*	3
Transport, peroxisomal	34	*Tetrahydrobiopterin metabolism*	9	*Arginine and proline metabolism*	2
Starch and sucrose metabolism	33	*Aminosugar metabolism*	8	*Hippurate metabolism*	2
Transport, endoplasmic reticular	29	*Vitamin D metabolism*	8	*N-glycan metabolism*	2
Transport, lysosomal	28	*Androgen and estrogen synthesis and metabolism*	7	*Protein formation*	2
Tryptophan metabolism	28	*Glycerophospholipid metabolism*	7	*Xenobiotics metabolism*	2
Vitamin A metabolism	28	*Pyrimidine catabolism*	7	*Alanine and aspartate metabolism*	1
Phenylalanine metabolism	23	*Arachidonic acid metabolism*	6	*Alkaloid synthesis*	1
Valine, leucine, and isoleucine metabolism	22	*Galactose metabolism*	6	*Biotin metabolism*	1
Eicosanoid metabolism	21	*Sphingolipid metabolism*	6	*Blood group synthesis*	1
Transport, golgi apparatus	20	*Steroid metabolism*	6	*CoA catabolism*	1
Bile acid synthesis	18	*Fructose and mannose metabolism*	5	*CoA synthesis*	1
Lysine metabolism	18	*NAD metabolism*	5	*Heme synthesis*	1
Ubiquinone synthesis	18	*Purine catabolism*	5	*Lipoate metabolism*	1
Fatty acid synthesis	14	*R group synthesis*	5	*Phosphatidylinositol phosphate metabolism*	1
Histidine metabolism	14	*Vitamin B6 metabolism*	5	*Propanoate metabolism*	1
Inositol phosphate metabolism	13	*Vitamin C metabolism*	5	*Vitamin B12 metabolism*	1
Cytochrome metabolism	12	*C5-branched dibasic acid metabolism*	4		

bioRxiv preprint doi: https://doi.org/10.1101/2024.07.24.604914; this version posted July 24, 2024. The copyright holder for this preprint (which was not certified by peer review) is the author/funder, who has granted bioRxiv a license to display the preprint in perpetuity. It is made available under aCC-BY 4.0 International license.

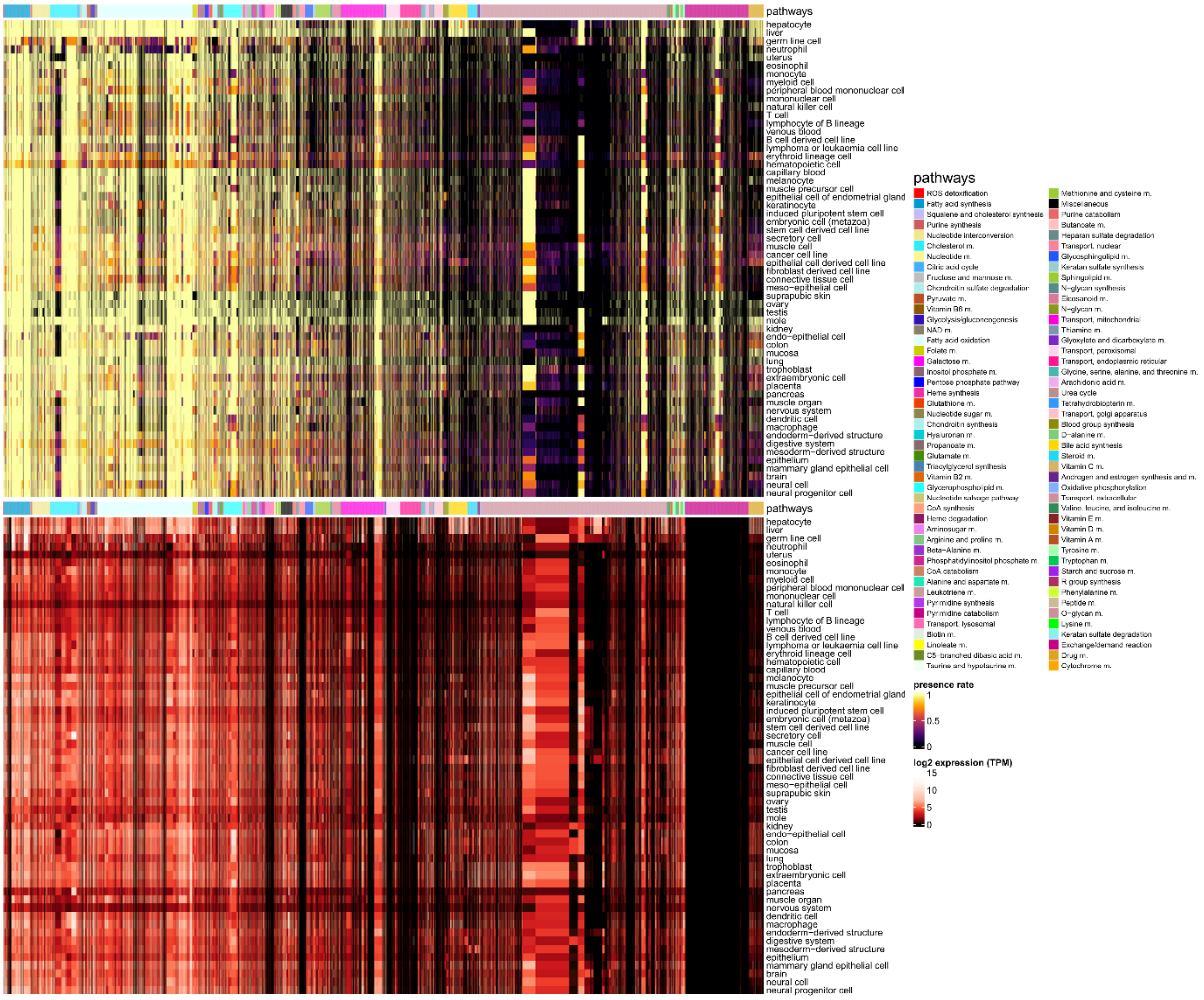

Supplementary Figure S3: The presence rate of the reactions across the 1,555 models corresponds to the gene expression mapped to Recon3D via the Gene Protein Reaction rules.

Supplementary Table S3: Core metabolic genes are enriched for genes implicated in splicing and mRNA processing and energy production, nucleotide, lipid metabolism and ROS metabolism.

GENE ONTOLOGY	OVERLAP	P-VALUE
CORE GENES		
MRNA SPLICING VIA SPLICEOSOME (GO:0000398)	148/211	3.16E-60
MRNA PROCESSING (GO:0006397)	145/214	3.71E-56
RNA SPLICING VIA TRANSESTERIFICATION REACTIONS WITH BULGED ADENOSINE AS NUCLEOPHILE (GO:0000377)	130/180	1.77E-55
TRANSLATION (GO:0006412)	142/234	2.11E-46
GENE EXPRESSION (GO:0010467)	164/296	2.11E-46
RIBOSOME BIOGENESIS (GO:0042254)	106/155	1.66E-41
RNA PROCESSING (GO:0006396)	113/183	8.30E-38
CYTOPLASMIC TRANSLATION (GO:0002181)	74/93	3.65E-36
RIBONUCLEOPROTEIN COMPLEX BIOGENESIS (GO:0022613)	81/118	1.06E-31

bioRxiv preprint doi: https://doi.org/10.1101/2024.07.24.604914; this version posted July 24, 2024. The copyright holder for this preprint (which was not certified by peer review) is the author/funder, who has granted bioRxiv a license to display the preprint in perpetuity. It is made available under aCC-BY 4.0 International license.

MACROMOLECULE BIOSYNTHETIC PROCESS (GO:0009059)	104/183	2.35E-30
RNA SPLICING (GO:0008380)	71/98	4.03E-30
PEPTIDE BIOSYNTHETIC PROCESS (GO:0043043)	93/158	9.88E-29
PROTEIN-RNA COMPLEX ASSEMBLY (GO:0022618)	89/150	7.60E-28
RIBOSOMAL SMALL SUBUNIT BIOGENESIS (GO:0042274)	61/84	6.70E-26
RRNA PROCESSING (GO:0006364)	68/101	7.91E-26
PROTEASOME-MEDIATED UBIQUITIN-DEPENDENT PROTEIN CATABOLIC PRO.	139/319	4.21E-25
NCRNA PROCESSING (GO:0034470)	63/100	1.63E-21
RRNA METABOLIC PROCESS (GO:0016072)	59/91	4.69E-21
METABOLIC CORE GENES		
METABOLISM R-HSA-1430728	210/2049	6.61E-133
CITRIC ACID (TCA) CYCLE AND RESPIRATORY ELECTRON TRANSPORT	64/163	7.18E-72
RESPIRATORY ELECTRON TRANSPORT, ATP SYNTHESIS BY CHEMIOSMOTIC COUPLING, HEAT PRODUCTION BY UNCOUPLING PROTEINS R-HSA-163200	53/112	1.35E-64
RESPIRATORY ELECTRON TRANSPORT R-HSA-611105	44/90	3.55E-54
COMPLEX I BIOGENESIS R-HSA-6799198	27/51	4.69E-34
METABOLISM OF NUCLEOTIDES R-HSA-15869	24/95	6.30E-21
METABOLISM OF LIPIDS R-HSA-556833	51/732	4.91E-18
CHOLESTEROL BIOSYNTHESIS R-HSA-191273	11/26	3.32E-12
INTERCONVERSION OF NUCLEOTIDE DI- AND TRIPHOSPHATES R-HSA-499943	11/28	7.98E-12
METABOLISM OF CARBOHYDRATES R-HSA-71387	26/285	1.52E-11
FORMATION OF ATP BY CHEMIOSMOTIC COUPLING R-HSA-163210	9/16	1.81E-11
PURINE RIBONUCLEOSIDE MONOPHOSPHATE BIOSYNTHESIS R-HSA-73817	7/10	8.26E-10
PYRUVATE METABOLISM AND CITRIC ACID (TCA) CYCLE R-HSA-71406	12/54	9.25E-10
NUCLEOTIDE BIOSYNTHESIS R-HSA-8956320	7/13	9.74E-09
CRISTAE FORMATION R-HSA-8949613	9/29	9.78E-09
ACTIVATION OF GENE EXPRESSION BY SREBF (SREBP) R-HSA-2426168	10/42	1.66E-08
METABOLISM OF VITAMINS AND COFACTORS R-HSA-196854	18/186	1.66E-08
FATTY ACID METABOLISM R-HSA-8978868	17/173	3.74E-08
PKMTS METHYLATE HISTONE LYSINES R-HSA-3214841	10/47	4.70E-08
METABOLISM OF WATER-SOLUBLE VITAMINS AND COFACTORS R-HSA-196849	14/122	1.30E-07
REGULATION OF CHOLESTEROL BIOSYNTHESIS BY SREBP (SREBF) R-HSA-1655829	10/55	2.16E-07
GLUCONEOGENESIS R-HSA-70263	8/32	4.47E-07
BIOSYNTHESIS OF N-GLYCAN PRECURSOR (DOLICHOL LLO) AND TRANSFER TO PROTEIN R-HSA-446193	11/77	4.99E-07
HEME BIOSYNTHESIS R-HSA-189451	6/14	6.47E-07
SYNTHESIS OF SUBSTRATES IN N-GLYCAN BIOSYTHESIS R-HSA-446219	10/63	7.11E-07
TP53 REGULATES METABOLIC GENES R-HSA-5628897	11/81	7.59E-07
BIOLOGICAL OXIDATIONS R-HSA-211859	17/218	8.13E-07
PHASE II - CONJUGATION OF COMPOUNDS R-HSA-156580	12/107	1.51E-06
MITOCHONDRIAL BIOGENESIS R-HSA-1592230	11/89	1.84E-06
METABOLISM OF PORPHYRINS R-HSA-189445	7/28	2.57E-06
CYTOPROTECTION BY HMOX1 R-HSA-9707564	9/57	2.92E-06
IRON UPTAKE AND TRANSPORT R-HSA-917937	9/59	3.85E-06

bioRxiv preprint doi: https://doi.org/10.1101/2024.07.24.604914; this version posted July 24, 2024. The copyright holder for this preprint (which was not certified by peer review) is the author/funder, who has granted bioRxiv a license to display the preprint in perpetuity. It is made available under aCC-BY 4.0 International license.

PYRUVATE METABOLISM R-HSA-70268	7/31	5.00E-06
METABOLISM OF STEROIDS R-HSA-8957322	13/153	9.50E-06
MITOCHONDRIAL FATTY ACID BETA-OXIDATION R-HSA-77289	7/36	1.40E-05
INSULIN RECEPTOR RECYCLING R-HSA-77387	6/26	2.83E-05
SYNTHESIS OF UDP-N-ACETYL-GLUCOSAMINE R-HSA-446210	4/8	4.54E-05
VITAMIN B5 (PANTOTHENATE) METABOLISM R-HSA-199220	5/17	5.33E-05
CELLULAR RESPONSE TO CHEMICAL STRESS R-HSA-9711123	13/182	5.80E-05
GLYCOSPHINGOLIPID METABOLISM R-HSA-1660662	7/45	5.92E-05
TRANSFERRIN ENDOCYTOSIS AND RECYCLING R-HSA-917977	6/31	7.45E-05
ASPARAGINE N-LINKED GLYCOSYLATION R-HSA-446203	16/282	8.72E-05
SPHINGOLIPID METABOLISM R-HSA-428157	9/89	9.47E-05
GLUCOSE METABOLISM R-HSA-70326	9/89	9.47E-05

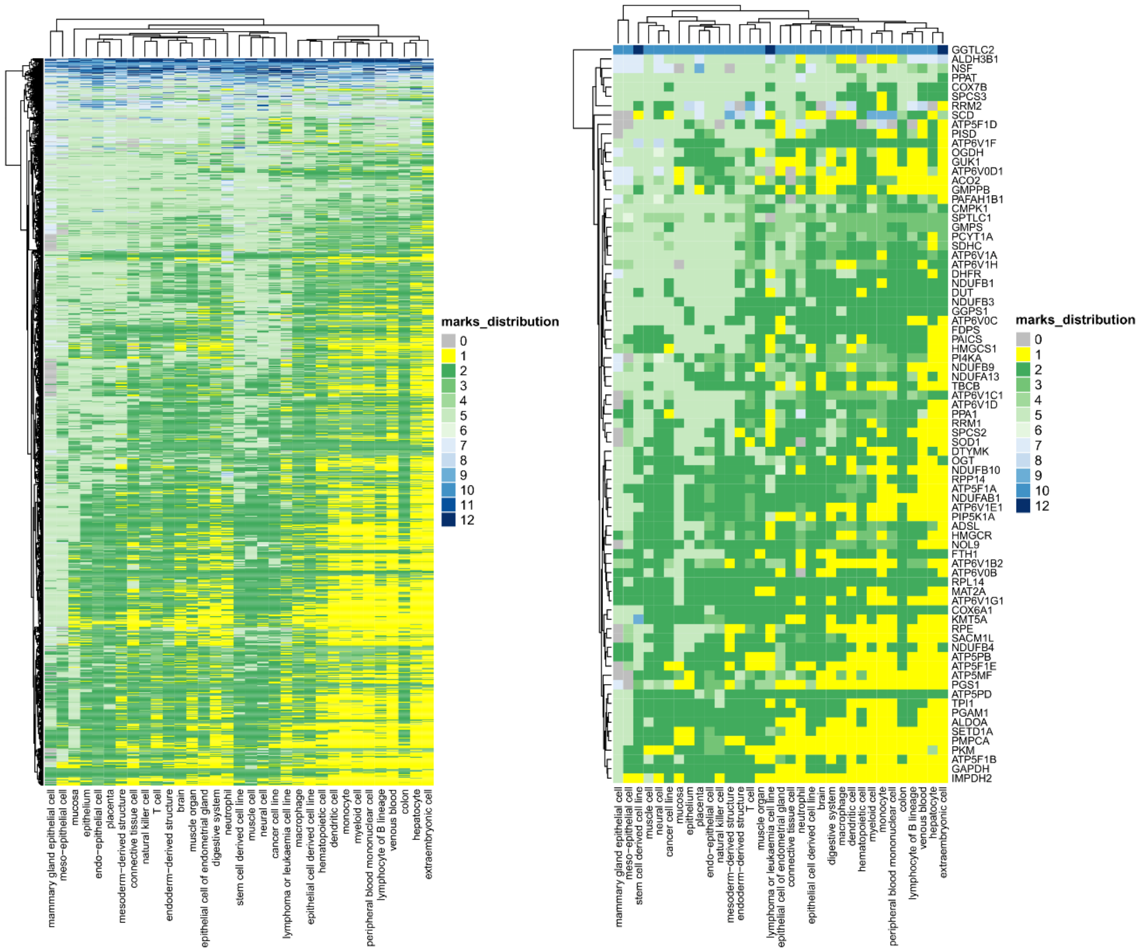

Supplementary Figure S4: Metabolic gene ChromGene States across cell types. 60% of common (left) and metabolic essential genes (right) were in inactive ChromGene states (above seven, in blue) across 33 cell types. The remaining genes were tagged with active ChromGene state (1-6, yellow and green) for most cell types. However, several genes, such as gamma-glutamyl transferase (GGTLC2) were associated with repressive marks in almost all cell types.

bioRxiv preprint doi: https://doi.org/10.1101/2024.07.24.604914; this version posted July 24, 2024. The copyright holder for this preprint (which was not certified by peer review) is the author/funder, who has granted bioRxiv a license to display the preprint in perpetuity. It is made available under aCC-BY 4.0 International license.

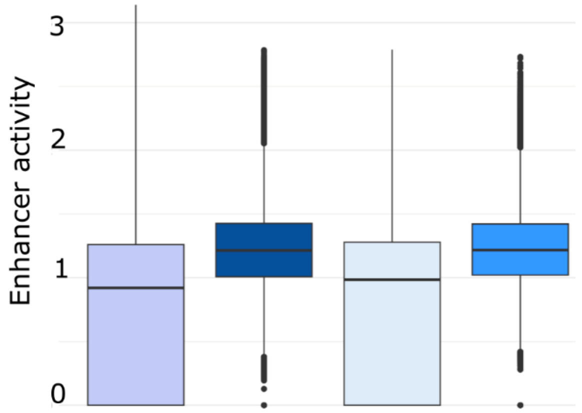

Supplementary Figure S5: Unique genes and unique metabolic genes enhancer activities.

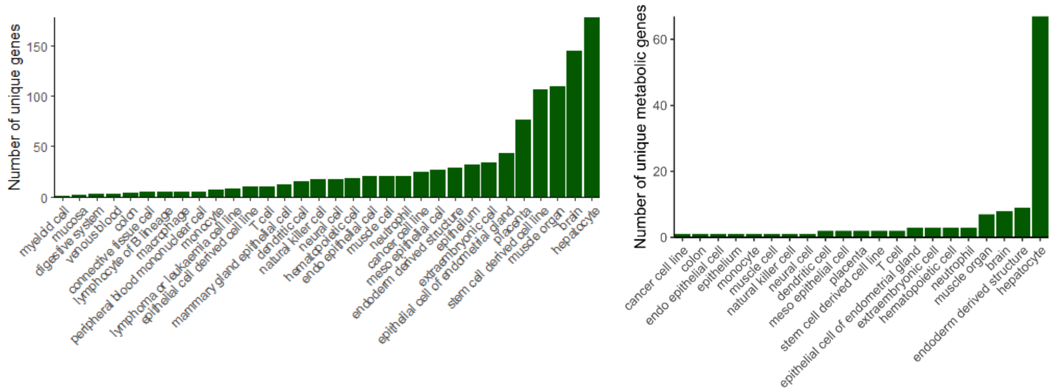

Supplementary Figure S6: Repartition of the unique (left) and unique metabolic genes across tissue and cell types (right)

Supplementary Table S4: Unique genes for hepatocyte, brain, and muscle organs are enriched for organ-matched gene ontology terms. Unique genes were defined by having an active consensus ChromGene state (states below 7) in one single tissue or cell type. No significant enrichments were found for colon and connective tissue cell line due to a limited number of genes (4, 5 genes). Similarly, no enrichments were found for cancer lines due to the substantial tissue heterogeneity of this category.

Tissue	unique genes	*Database*	Term	Overlap	Adj. p-value
Hepatocyte	177	KEGG	Steroid hormone biosynthesis	12/61	2.03E-11
			Complement and coagulation cascades	13/85	2.97E-11
			Drug metabolism	13/108	4.64E-10
			Metabolism of xenobiotics by cytochrome P450	11/76	1.80E-09
			Bile secretion	10/90	1.54E-07
			Retinol metabolism	9/68	1.56E-07
			Chemical carcinogenesis	13/239	3.41E-06
			Linoleic acid metabolism	5/29	7.17E-05

bioRxiv preprint doi: https://doi.org/10.1101/2024.07.24.604914; this version posted July 24, 2024. The copyright holder for this preprint (which was not certified by peer review) is the author/funder, who has granted bioRxiv a license to display the preprint in perpetuity. It is made available under aCC-BY 4.0 International license.

			Arachidonic acid metabolism	6/61	2.03E-04
			Maturity onset diabetes of the young	4/26	8.01E-04
			Cholesterol metabolism	5/50	8.01E-04
Brain	144	Reactome	Receptor-type Tyrosine-Protein Phosphatases	5/20	4.87E-05
			Protein-protein Interactions at Synapses	7/88	3.21E-04
			Neuronal System	12/386	1.50E-03
Muscle Organ	109	Reactome	Muscle Contraction	10/196	2.97E-05
			Striated Muscle Contraction	5/33	1.15E-04
			Myogenesis	4/29	0.0014
Placenta	76	Descartes	Trophoblast giant cells in Placenta,	17/89	4.20E-23
			CSH1 CSH2 positive cells in Adrenal	16/272	1.49E-13
Neutrophils	21	Reactome	Neutrophil Degranulation	9/468	1.43E-08
		Reactome	Innate Immune System R-HSA-	9/1035	6.88E-06
		Reactome		10/1943	1.01E-04
Dendritic cell	15	PanglaoDB _Augmented	Dendritic Cells	2/199	0.06

Supplementary Table S5: Unique metabolic genes for hepatocyte, brain, and muscle are enriched for organ-matched gene ontology terms. Unique genes specific to one tissue were defined by having a consensus ChromGene below 7 in one single tissue or cell type.

bioRxiv preprint doi: https://doi.org/10.1101/2024.07.24.604914; this version posted July 24, 2024. The copyright holder for this preprint (which was not certified by peer review) is the author/funder, who has granted bioRxiv a license to display the preprint in perpetuity. It is made available under aCC-BY 4.0 International license.

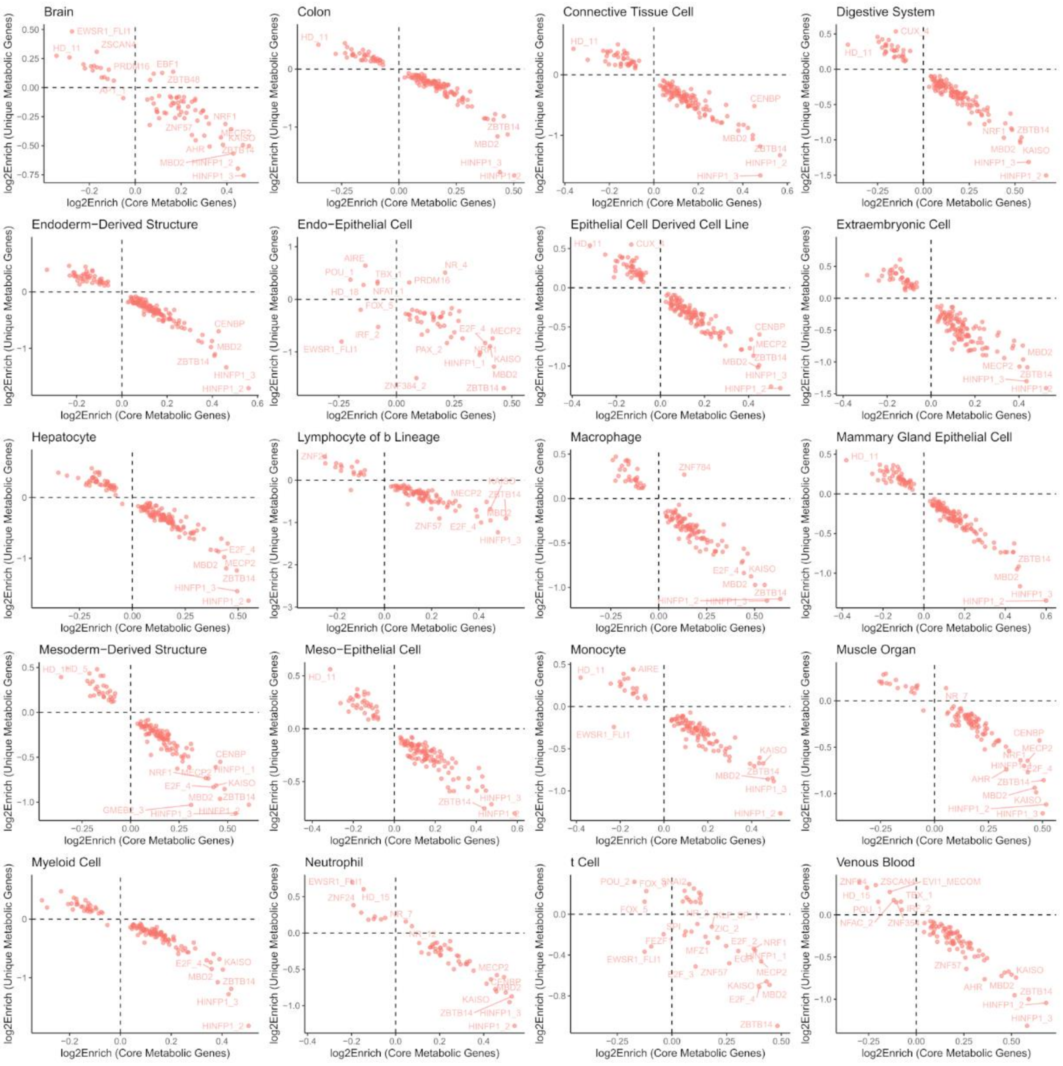

Supplementary Figure S7: Correlation between TF enrichments in enhancers linked to unique metabolic genes vs core metabolic genes.

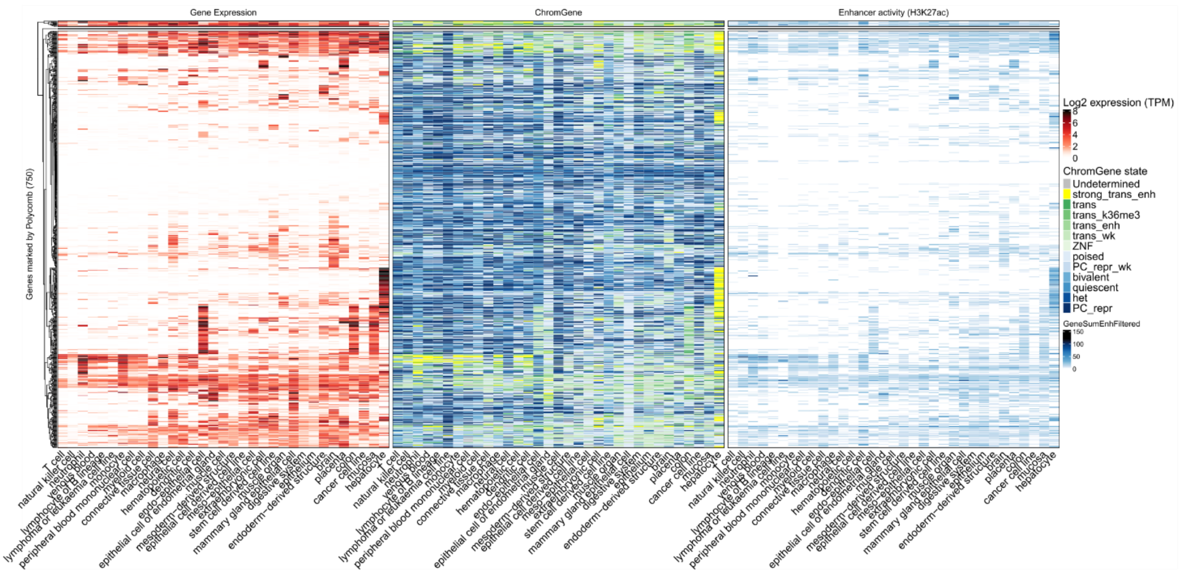

bioRxiv preprint doi: https://doi.org/10.1101/2024.07.24.604914; this version posted July 24, 2024. The copyright holder for this preprint (which was not certified by peer review) is the author/funder, who has granted bioRxiv a license to display the preprint in perpetuity. It is made available under aCC-BY 4.0 International license.

Supplementary Figure S8: Expression, ChromGene states and sum enhancer and gene interactions for polycomb-associated genes.

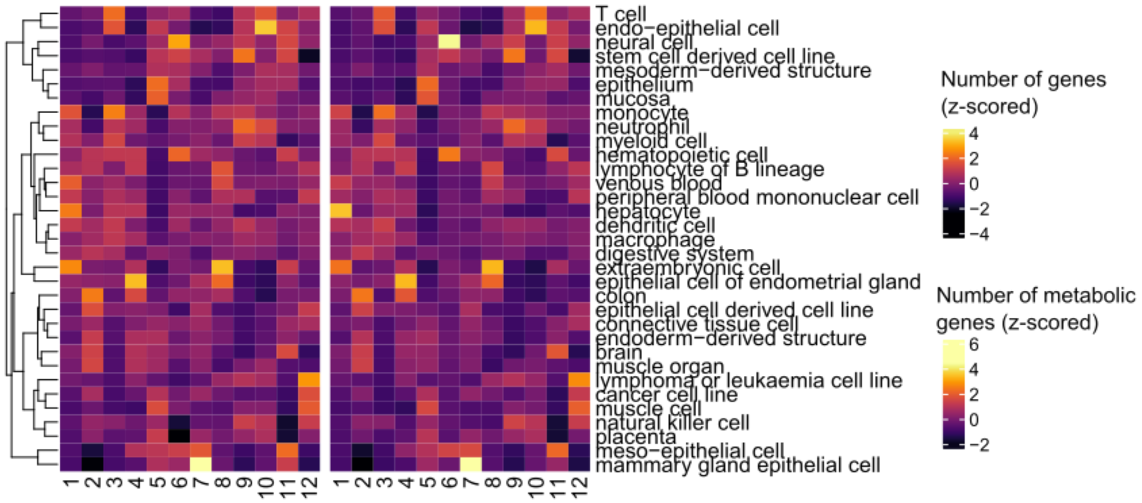

Supplementary Figure S9: Number of genes associated with the twelve ChromGene states. Chrome 1-6 are linked to an active state, 7 stand for poised and 8-12 to repressive marks.

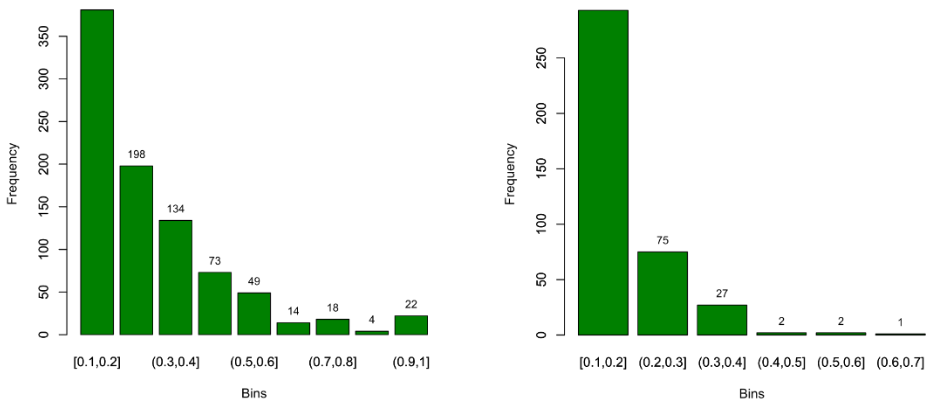

Supplementary Figure S10: Number of pathway-cell type combinations having a presence rate between 0.1-0.7 for ChromGene 1 (left) and between 0.1-1 for ChromGene 12 (right).

Supplementary Table S8: Number of cell types with a presence rate above 0.6 for strongly transcribed genes with enhancer marks H3K4me1 and H3K27ac (ChromGene State 1)

Pathways	Number of cell types	Number of reactions
Keratan sulfate synthesis	9	22
Lipoate metabolism	9	1
Alkaloid synthesis	5	1
Keratan sulfate degradation	3	76
Glycolysis/gluconeogenesis	3	42

bioRxiv preprint doi: https://doi.org/10.1101/2024.07.24.604914; this version posted July 24, 2024. The copyright holder for this preprint (which was not certified by peer review) is the author/funder, who has granted bioRxiv a license to display the preprint in perpetuity. It is made available under aCC-BY 4.0 International license.

Fructose and mannose metabolism	3	23
Vitamin B6 metabolism	3	11
Hyaluronan metabolism	3	5
Nucleotide metabolism	3	1
Fatty acid oxidation	1	961
Cholesterol metabolism	1	242
Fatty acid synthesis	1	239
Folate metabolism	1	59
Pyruvate metabolism	1	32
Glutamate metabolism	1	16
Cytochrome metabolism	1	15
Purine synthesis	1	15
Glyoxylate and dicarboxylate metabolism	1	14
Alanine and aspartate metabolism	1	13
Triacylglycerol synthesis	1	9
Leukotriene metabolism	1	8
ROS detoxification	1	7
Squalene and cholesterol synthesis	1	5
Butanoate metabolism	1	3
Limonene and pinene degradation	1	3
Nucleotide sugar metabolism	1	1

Supplementary Table S9: Number of cell types with a presence rate above 0.3 for Polycomb-mediated repressed genes (ChromGene State 12)

**Pathways**	**Number of cell types**	**Number of reactions**
Cytochrome metabolism	12	15
Vitamin D metabolism	4	13
Alanine and aspartate metabolism	3	13
Steroid metabolism	3	90
Bile acid synthesis	2	185
D-alanine metabolism	2	3
Androgen and estrogen synthesis and metabolism	1	26
Blood group synthesis	1	47
Butanoate metabolism	1	3
Glycine, serine, alanine, and threonine metabolism	1	47
O-glycan metabolism	1	18
Triacylglycerol synthesis	1	9
